# COVID-19 pandemic and the widening oral health inequality in Nigeria

**DOI:** 10.11604/pamj.2022.41.40.26549

**Published:** 2022-01-17

**Authors:** Toluwani Ifeoluwa Oluwatola, Oluwapelumi Micheal Olowookere, Morenike Oluwatoyin Folayan

**Affiliations:** 1 Lagos State Health Management Agency, Lagos, Nigeria,; 2 Beachland Specialist Hospital, Arepo Ogun State, Nigeria,; 3 Faculty of Dentistry, Obafemi Awolowo University, Ile Ife, Nigeria

**Keywords:** COVID-19, oral health, Nigeria, teledentistry, health insurance

## Abstract

The inequality in access to oral health care in Nigeria is driven by the low numbers of trained health care workers, disproportionate distribution of oral health facilities, low level of oral health awareness, and the challenge associated with out-of-pocket expenditures. The COVID-19 pandemic disrupted oral health care delivery, access to oral health care services, thereby further entrenched inequality by increasing the out-of-pocket expenditure for health due to COVID-19 associated increased cost of medical services; high risk of worsening oral health care needs by patients who have routine and special oral health care needs; increased risk for oral health care needs by persons worse affected by COVID-19; and the high risk for general health problems by those whose access to routine and special health care needs were disrupted by the pandemic. The pandemic has however, also created opportunities to reduce the inequalities in the oral health care sector through adoption of teledentistry; integrated oral and general health care; improving oral health insurance coverage for the informal sector; and increasing public financing for health.

## Essay

Many oral diseases disproportionately affect people of lower socioeconomic status; and they are highly prevalent in low- and middle-income countries. These countries are also less able to institute oral health diseases mitigation and control plans because they lack the data, human and infrastructure resources needed to plan and address the growing oral health needs; and also have constraints in offering citizens the financial support to prevent huge out-of-pocket oral health care expenses [1].

Nigeria is a lower-middle-income country with unmet oral health care needs. The prevalence of untreated dental caries ranges from 23.1% in urban areas to 35.5% in rural areas [2,3]. The country has only 7,320 trained dental workers (dentists and dental therapists, nurses, and hygienists) providing clinical oral healthcare to its over 200 million population [4,5]. Oral health facilities are disproportionately located in the southern part of the country (about half of private oral health providers are in Lagos State, which is one of the 36 states in Nigeria), and only 20% of the oral health workforce practice in the rural areas where about 60% of the population reside [4].

People face multiple structural barriers to access to oral health care in Nigeria - only 26.4% of Nigerians have had at least one dental visit [6], one of which is the cost. Dental treatment is expensive [7], and about 90% of Nigerians pay out-of-pocket for health care services [8]. For the few who have health insurance, the dental services covered by insurance packages are very limited. The inability of Nigerians to have health insurance coverage have limited their use of oral health care services, with people with low socio-economic status being worse affected.

A second barrier is the distance between the residential area and the location of dental clinics. People who live farther away from dental facilities are less likely to seek oral health care. People residing in rural areas and poorer neighborhoods also are less able to access dental care service delivery points more often located in urban areas and affluent neighborhoods [9]. Though the use of mobile clinics to provide outreach oral health services can mitigate this challenge, mobile oral health care clinics are no longer accessible in the country. A third barrier to access to oral health care in Nigeria is the poor awareness, knowledge and attitude to oral health care. Oral health is more often sought for curative rather than preventive purposes. There are very few oral health informed behavior changes attributable to the low level of education and socio-economic status of the majority of Nigerians: individuals with lower educational levels and socio-economic status have less knowledge and poorer attitude to oral health, and they use oral health facilities less often than do those with higher educational and socioeconomic status [10].

**COVID-19 and oral health in Nigeria:** these and other structural barriers create unequal opportunities for Nigerians to have oral health thereby widening the existing inequalities for socially disadvantaged persons. This inequality in access to care has implications for people´s physical, emotional, psychological, and socioeconomic well-being. Sadly, COVID-19 worsens the social disparity in oral health care in Nigeria. Nigeria is in its fourth wave of the COVID-19 pandemic with 234,709 documented infections and 2,991 COVID-19 related deaths [11]. During the first wave of the pandemic, one of the government´s responses was the lockdown of the economy and restriction of movement between states and countries from March 30^th^, 2020, to May 4^th^, 2020. During this lockdown, about 80% of private dental clinics in the country were shut [12], and the public dental clinics were open only to emergency care, in line with the federal government´s COVID-19 protocol for oral health care [13]. Many dental clinics reopened for business during the phased easing of the lockdown that began on May 4^th^, 2020, but clinics are not allowed to manage patients with COVID-19 symptoms; symptomatic and asymptomatic patients with COVID-19 infections are expected to be referred to designated COVID-19 management centers in any of the 37 states in Nigeria [14]. The COVID-19 management centers do not have oral health care strategies included in their patient care protocol. This has implications for overall health of the patients, and further entrenching oral health care inequalities in the country.

First, COVID-19 induces diabetes mellitus [15] and renal diseases [16] are linked with oral health problems: uncontrolled diabetes mellitus and chronic renal failure increase the risk for chronic periodontitis [17]. Ironically, the populations vulnerable to COVID-19 - adults, those with low socioeconomic status and low-literacy, those in rural areas, and those who are uninsured [18] - are also those with increased risk for oral diseases many of which are unable to access oral health care.

Second, during the lockdown, movements were also restricted affecting the use of the hospitals and clinics. Also, outreach education and dental health programs to remote areas of the country were suspended. COVID-19-induced fear and anxiety may also increase the prevalence of smoking, which has severe oral-health consequences. Even after the lockdown patients had continued to stay away from the dental clinic because of perceived risk for contracting COVID-19 in hospital settings [19]. These may have consequences. The poor access of remote communities to oral health programs may worsen the oral health plight of people in these communities [14]. Also, patients with routine and special oral health care needs may have to deal with deterioration of their oral health, and worsening of their medical health, such as the risk of developing insulin resistance and type 2 diabetes in patients with chronic periodontitis [17].

The link between oral health and general health is mainly inflammation induced. COVID-19 is an inflammatory disease, with molecular mimicry and associated chronic oxidative stress leading to autoimmune reactions. The release of pro-inflammatory cytokine also triggers other inflammatory diseases, such as diabetes mellitus and chronic periodontitis. COVID-19-related stress and anxiety-induced smoking and alcohol intake are associated with destructive periodontal disease, chronic respiratory disease, and systemic changes in inflammatory cytokine levels and immune-modulator globulins, leading to the deterioration of chronic inflammatory conditions [20]. Empirical studies on the magnitude of these proximal and distal factors on oral health in general, and the oral health of Nigerians specifically, are limited but are highly needed for the development of oral health care guidance. Third, the heightened food insecurity resulting from the lockdown [21], may increase the risk for malnutrition in many children, with attendant oral health consequences, such as noma, acute necrotizing ulcerative gingivitis, and caries. These lesions were prevalent in Nigeria before COVID-19, and they likely will be more frequent as result of responses to the pandemic.

Sadly, with this prospect for increased oral health care needs is the associated capital flight that makes it costlier to procure oral health care services out-of-pocket. The cost of medical services had surged by 15.10% year on year since the pandemic began [22]. The income of Nigerians who work in the informal sector was severely hampered by the lockdown worsening their financial security status.

**New norms for oral health care in Nigeria post pandemic:** the Nigeria economy has reopened, and things are gradually settling into a new normal. The oral health care system in Nigeria will also need to adopt new normal statuses. Table 1 provides a summary of recommendations on ways to harness the opportunities created by the COVID-19 pandemic to reduce the inequalities associated with oral health care access post. A first new normal that can be created by the pandemic is the adoption of teledentistry. Teledentistry offers an innovative, cost-effective opportunity to overcome the challenges of inadequate dental personnel and the distance between dental personnel and potential patients in rural and remote communities [23]. Teledentistry can be used to assess, triage, diagnose, manage, and follow patients via a telephone or video link. Nigeria has a high internet penetration and mobile subscribers [24] making teledentistry hold the potential for bridging the gap in service access. Public-private partnership between entrepreneurial providers of telemedicine and public health providers can help scale up teledentistry services across the country, with focus on delivery in remote and rural areas.

**Table 1:** summary of key recommendations

Challenges	Proposed solution
Poor access to oral health care due to inadequate dental personnel and the distance between dental personnel and potential patients in rural and remote communities	Adoption of teledentistry
Neglect of oral health care needs of vulnerable populations during pandemics	Integrated oral and general health care
High cost of dental care service	Expand access of the informal work sector to health insurance coverage
Poor budgetary expenditure on health	Increase domestic resources for health

A second new normal should be the integration of oral health into general health care. During the lockdown, policymakers made concerned effort to reduce the impact of the pandemic access to reproductive, maternal, newborn and child healthcare; mental healthcare; HIV and tuberculosis care [25]. There was little public discussion and debate about the pandemic induced oral health morbidities. Incorporating oral health into existing other health programs is a cost-saving approach since oral health is linked to general health. The draft 2020 national oral health policy and the 2021-2025 national health policy promotes integrated health. The events of the pandemic highlights the need to facilitate the implementation of these policies. Instituting mechanisms to promote integrated oral and general health programs during the COVID-19 pandemic will likely help the country´s preparedness efforts for future pandemics.

A third new normal should be the drive to improve the oral health insurance coverage for the informal work sector. Oral health care constitutes about 5% of the total health expenditure, and it is responsible for about 20% of out-of-pocket expenditure [7]. Though the draft 2020 national oral health policy promotes preventative dental care, the national health insurance scheme is covers a lot more curative oral health care [26]. The post COVID-era should improve the access of Nigerians to oral health care through multiple mechanisms that will reduce their risk of being impoverished from oral health expenses. A tax-based, non-contributory health financing model can be a useful tool in achieving this new normal [27].

A fourth new normal should be an increase in the healthcare budgetary allocation (and through that oral healthcare budgetary allocation) by the government of Nigeria. In the 2020 budget, $1.11 billion was allocated for both capital and recurrent expenditure on health, a per capita allocation of only $5.53 [28]. Budgetary allocation to health continues to be below 5% of the national budget despite the country being a signatory to the 2001 Abuja declaration that pledged 15% of the annual budget to health. Public health financing needed to achieve universal health coverage, a tool for countries to reverse inequalities in oral health care access. Healthcare financing can be improved if the government becomes more accountable and efficient with its capital expenditure, and if it explores alternative sources of income to increase the fiscal space for health. This includes exploring the market-systems approach to promote public and private health sector engagement. The market-systems approach focuses on interventions that modify the incentives and behavior of public, private, formal, and informal health care providers to ensure lasting and large-scale benefits to poor people [29].

In conclusion, the COVID-19 pandemic has had severe impact on the access of a large segment of the Nigerian population to oral health care, especially those who are more vulnerable to poor oral health, thereby worsening the existing inequality in access to oral health care in Nigeria. The response to the pandemic can be harnessed to create ‘new normals´ that will reduce out-of-pocket oral health expenses and facilitate access of all citizens to oral health care.
